# Metal- and antibiotic-resistant heterotrophic plate count bacteria from a gold mine impacted river: the Mooi River system, South Africa

**DOI:** 10.1007/s11356-022-24015-3

**Published:** 2022-11-30

**Authors:** Janita Bosch, Carlos Bezuidenhout, Roelof Coertze, Lesego Molale-Tom

**Affiliations:** Unit for Environmental Sciences and Management, Microbiology, North-West University, Potchefstroom Campus, Private Bag X6001, Potchefstroom, 2520 South Africa

**Keywords:** Antimicrobial resistance, Heavy-metal tolerance, Co-occurrence, MAR phenotypes

## Abstract

The Wonderfonteinspruit, South Africa, is highly impacted by a century of gold mining activities. The aim of this study was to investigate the physico-chemical properties of the Wonderfonteinspruit and the receiving Mooi River system, the levels of antimicrobial (metals and antibiotics) resistance characteristics and heterotrophic bacteria levels in these water systems. Various physico-chemical parameters were determined. R2A agar and R2A agar supplemented with antimicrobials were used to enumerate heterotrophic bacteria. Morphologically distinct antimicrobial-resistant isolates were purified and screened for antibiotic susceptibility by a disc diffusion method. Selected isolates were identified, and minimum inhibitory concentration ranges determined. Among the antimicrobial resistant isolates, 87% were resistant to at least one antibiotic. Of these, almost 50% were resistant to more than 3 antibiotic classes. A large proportion was resistant to all 7 antibiotics tested. Phyla detected were Proteobacteria, Firmicutes and Bacteriodetes. High MIC levels for metals and antibiotics were detected among all the genera. Results demonstrate potential impacts of physico-chemical properties on levels of antimicrobial-resistant bacteria. Metal-resistant bacteria were also resistant to multiple antibiotics, suggesting that metal pollution from mining may be responsible for co-selection and maintenance of antibiotic-resistant bacteria in this aquatic system.

## Introduction

Over the last decade, industrialisation and globalization have caused accidental surface water heavy metal contamination in various countries (Squadrone 2020). Metal-contaminated source waters, even if exposure is for a short period, become severely deteriorated due to the shock loading of the heavy metal(s) (Zhang et al. 2020). Global studies have suggested that metal contamination in the environment can directly select for metal resistance and co-select for antibiotic resistance (Martinez 2009; Bezuidenhout et al. 2017; Sun et al. 2021). Recently, a study by Lear et al. (2022) also reported that metal-contaminated environments can select for virulence factors. Additionally, Zhang et al. (2020) state that heavy metals have the ability to alter the antibiotic resistome of contaminated water bodies. It has become evident, in the last decade, that environments such as surface water systems harbor metal-resistant and antibiotic-resistant bacteria (Squadrone 2020; Truong et al. 2021). More so, the presence of metal-resistant and antibiotic-resistant bacteria in water has raised significant concerns (Squadrone 2020; Anand et al. 2021), while recent studies have started to identify multiple resistant genes associated with heavy metal impacted environments (Rajasekar et al. 2022; Yi et al. 2022). Nonetheless, information on the impacts of the physico-chemical properties of water on the metal and antibiotic resistance of bacteria is still lacking in South Africa. This is concerning when considering the health impacts that the natural environment and water sources have on all forms of life (Chudobova et al. 2014; Irannezhad et al. 2022). Additionally, this concern becomes more apparent when considering the use of surface water in South Africa for various anthropogenic activities such as irrigation, livestock watering and recreation (Molale & Bezuidenhout 2016).

A century of gold mining activities on the Witwatersrand of South Africa has severely impacted the Wonderfonteinspruit which converges with the Mooi River upstream from the town Potchefstroom (Coetzee et al. 2006). Tlokwe municipality (now JB Marks Municipality) regularly measures levels of heavy metals in the Mooi River catchment. Metal concentrations (Table 2) of sites, relevant to the current study, were thus obtained from the municipality and indicated that metal concentrations in this system were between 2.5 and 1067 times the minimum co-selective concentrations (Seiler and Berendonk 2012). Furthermore, both these systems are impacted by agriculture, urbanization and rural settlements (Van der Walt et al. 2002; Koekemoer et al. 2021).

Concerns of the impacts of mining on the metal concentrations in the Mooi River have been a topic of discussion for many years (van der Walt et al. 2002). Jordaan and Bezuidenhout (2016) found that some of the water quality parameters measured in the Mooi River exceeded the resource water quality objectives (RWQO’s; Table 1) set out, specifically, for the Mooi River catchment (DWAF 2009). Furthermore, they detected the presence of a diverse bacterial community by using next generation sequencing and described the impacts of the physico-chemical environment on the bacterial community composition. Due to the culture-independent nature of their study, metal and antibiotic resistance among bacteria could not be studied. However, Molale and Bezuidenhout (2016) demonstrated that several enterococci species isolated from the Mooi River were resistant to multiple antibiotic classes. Furthermore, Carstens et al. (2014) also demonstrated that multiple antibiotic resistance was common among heterotrophic bacteria (HPC) from boreholes in the Mooi River system. Also, although several studies have assessed the co-occurrence of metal-tolerant and antibiotic resistant bacteria globally, only few have been conducted in South Africa (Jardine et al. 2019). Thus, this study is the first to report the presence of metal-tolerant and antibiotic-resistant HPC bacteria in a River in the North West Province, South Africa. Also, the present study strives to bridge the gap of limited knowledge of the state of the water resources in the North West Province and state of antibiotic and metal resistance of bacteria in the aquatic system impacted by mining, agriculture and urbanization. Such data are important since heterotrophic bacteria, particularly in pollution-impacted aquatic systems may adapt to these conditions in order to survive. This survival may include the acquisition or mobilization of genetic elements including genes on plasmids and transposable elements. Previous studies have found that co-resistance for multiple antibiotics in bacteria could at times be linked to metals such as copper, lead, iron and zinc (Seiler and Berendonk 2012; Sun et al. 2021).

**Table 1:** Physico-chemical variables and antimicrobial-resistant HPC profiles at sites surrounding the Mooi River and Wonderfonteinspruit confluence. Values exceeding the resource water quality objectives (RWQO) are indicated in italics and bold

			WFS 1		WFS 2		MR-BC 1		MR-BC2			MR-AC1			MR-AC 2			
Parameter	Unit	RWQO	March	July	March	July	March	July		March	March		July		March		July	
**pH**		8.0	8.34 ± 0.01	8.31 ± 0.01	8.35 ± 0.01	8.23 ± 0.01	8.18 ± 0.01	8.40 ± 0.01		7.50 ± 0.07	7.78 ± 0.02			8.05 ± 0.01		8.13 ± 0.00		8.50 ± 0.01
**Temp**	**°C**	N/A	22.10 ± 0.00	14.90 ± 0.08	21.57 ± 0.05	11.03 ± 0.05	22.20 ± 0.08	14.33 ± 0.12		27.77 ± 0.05	20.50 ± 0.08			12.60 ± 0.45		21.68 ± 0.25		14.10 ± 0.08
**TDS**	**mg/L**	370.0	***639.00*** ± 4.55	***838.00*** ± 1.63	***585.00*** ± 17.91	***846.00*** ± 2.94	***274.00*** ± 0.00	354.00 ± 1.41		***430.07*** ± 0.95	***504.33*** ± 3.09			***603.00*** ± 2.16		***426.33*** ± 9.39		***577.67*** ± 2.62
**DO**	**mg/L**	N/A	7.43 ± 0.12	8.33 ± 0.37	7.13 ± 0.26	14.87 ± 0.65	7.23 ± 0.95	10.90 ± 0.29		6.77 ± 0.12	6.87 ± 0.52			9.77 ± 0.19		6.30 ± 0.16		10.60 ± 0.33
**NO**_**3**_^**−**^	**mg/L**	0.3	0.30 ± 0.00	0.00 ± 0.00	0.20 ± 0.00	0.00 ± 0.00	***0.50*** ± 0.20	0.20 ± 0.10		***51.25*** ± 0.85	***1.05*** ± 0.05			***1.60*** ± 0. 10		***0.45*** ± 0.05		***0.85*** ± 0.25
**PO**_**4**_^**2−**^	**mg/L**	0.4	NA	***7.24*** ± 0.18	NA	***3.40*** ± 0.05	NA	***0.55*** ± 0.23		NA	NA			***0.34*** ± 0.05		NA		***0.57*** ± 0.07
**SO**_**4**_^**2−**^	**mg/L**	75.0	***150.00*** ± 0.38	***122.50*** ± 1.50	***90.50*** ± 0.50	***109.00*** ± 5.00	1.50 ± 0.50	***75.00*** ± 1.50		0.00 ± 0.00	***89.00*** ± 1.00			***93.50*** ± 2.50		***85.00*** ± 2.00		***90.00*** ± 1.00
**COD**	**mg/L**	N/A	2.00 ± 1.00	7.50 ± 0.50	20.50 ± 5.50	9.00 ± 0.00	22.50 ± 4.50	3.00 ± 0.00		45.50 ± 3.01	8.00 ± 2.00			0.00 ± 0.00		7.00 ± 1.00		0.00 ± 0.00
**HPC**	**CFU/Ml**	N/A	2.75 × 10 ^6^	1.31 × 10 ^5^	1.53 × 10 ^6^	9.10 × 10 ^4^	n/a	2.81 × 10 ^4^		2.30 × 10 ^6^	1.47 × 10 ^5^			9.66 × 10 ^3^		1.02 × 10 ^5^		6.66 × 10 ^2^
*HPC- AMP	**CFU/Ml**	N/A	2.62 × 10 ^3^	1.55 × 10 ^3^	4.72 × 10 ^3^	5.20 × 10 ^2^	n/a	1.50 × 10 ^1^		1.27 × 10 ^5^	4.31 × 10 ^3^			9.50 × 10 ^1^		2.63 × 10 ^3^		4.00 × 10 ^1^
^**$**^**HPC–Cu**	**CFU/Ml**	N/A	1.14 × 10 ^3^	1.53 × 10 ^2^	1.17 × 10 ^2^	N/D	n/a	N/D		2.83 × 10 ^3^	N/D			N/D		N/D		N/D
^**$**^**HPC-Fe**	**CFU/Ml**	N/A	2.38 × 10 ^3^	5.15 × 10 ^2^	1.04 × 10 ^3^	2.50 × 10 ^2^	n/a	9.70 × 10 ^1^		2.20 × 10 ^5^	1.00 × 10 ^4^			2.50 × 10 ^2^		1.78 × 10 ^3^		7.50 × 10 ^1^
^**$**^**HPC-Pb**	**CFU/Ml**	N/A	1.10 × 10 ^5^	2.58 × 10 ^3^	6.42 × 10 ^3^	1.04 × 10 ^3^	n/a	7.70 × 10 ^1^		2.80 × 10 ^5^	2.00 × 10 ^4^			2.93 × 10 ^2^		2.09 × 10 ^5^		3.00 × 10 ^1^
^**$**^**HPC-Zn**	**CFU/Ml**	N/A	1.08 × 10 ^3^	1.08 × 10 ^2^	2.80 × 10 ^2^	6.50 × 10 ^1^	n/a	N/D		4.15 × 10 ^4^	7.40 × 10 ^2^			6.70 × 10 ^1^		1.25 × 10 ^2^		N/D

*RWQO*, Resource Water Quality Objectives; *WFS*, Wonderfonteinspruit; *MR BC*, Mooi River Before Confluence; *MR AC*, Mooi River After Confluence; *TDS*, Total Dissolved Solids; *DO*, Dissolved Oxygen; *COD*, Chemical Oxygen Demand; *N/A*, not applicable; *n/a*, not available; *N/D*, none determined; *HPC*, Heterotrophic Plate Count; *CFU*, Colony Forming Units; *AMP*, Ampicillin. *HPC-AMP, supplemented with 100 µg/Ml; ^*$*^*HPC-metal*, supplemented with 1 Mm metal. *Cu*, copper; *Fe*, iron; *Pb*, lead; *Zn*, zinc

The objectives of the present study were thus to (i) determine how the physico-chemical properties of the water impacts the levels and characteristics of heterotrophic bacteria, (ii) if co-occurrence of antibiotic and metal-resistant bacteria was observed among these bacteria from the Mooi River system, (iii) to identify multiple antibiotic-resistant isolates and (iv) to determine minimum inhibitory concentrations (MIC) levels of certain antibiotics to identified isolates.

## Materials and methods

### Site description

**Fig. 1:**
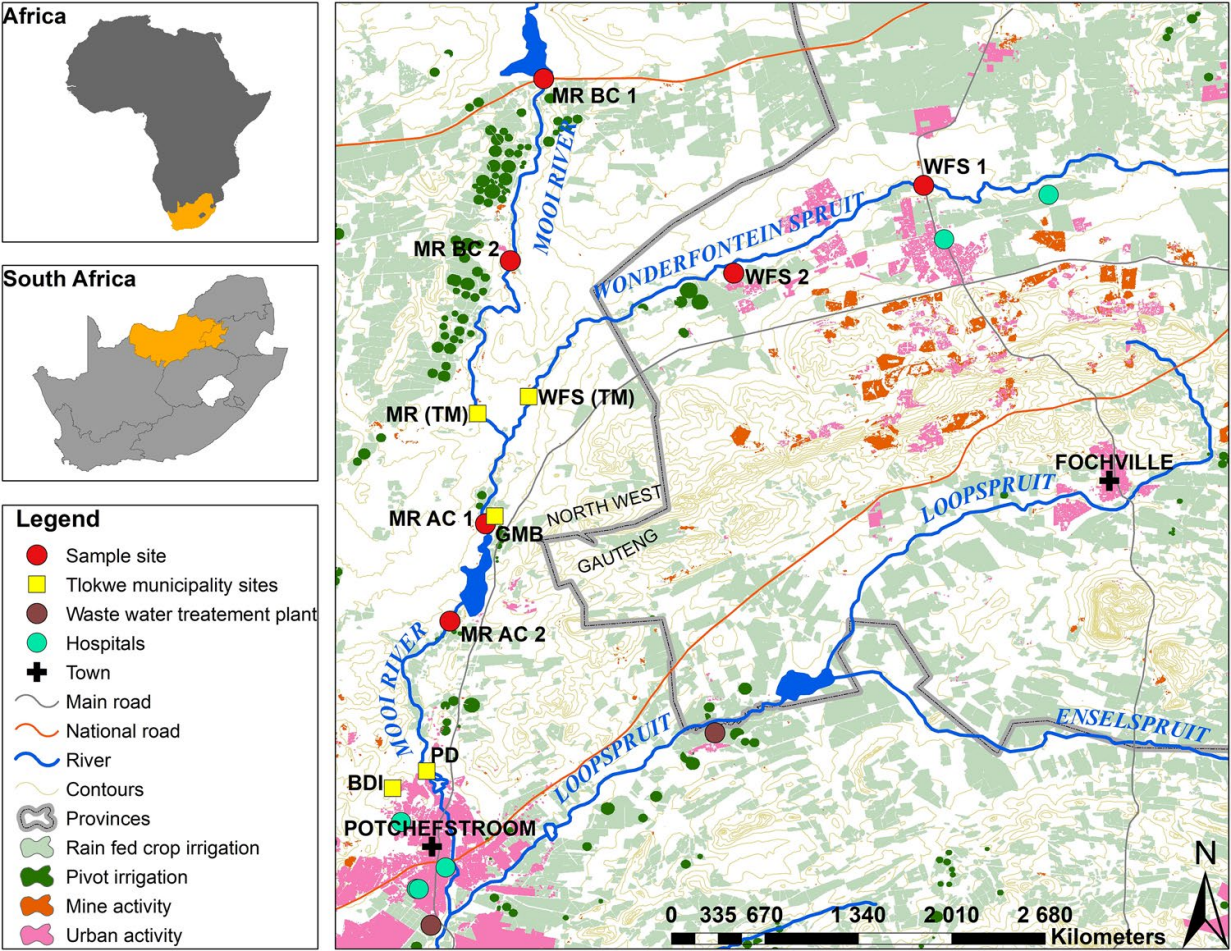
Map indicating the specific sites of the current study and the sites at which Tlokwe municipality measured heavy metal concentrations**.** MR — Mooi River, WFS — Wonderfonteinspruit, BC — Before Confluence, AC — After Confluence, (TM) — of Tlokwe Municipality, GMB — Gerhard minnebron, PD — Potchefstroom dam

Figure 1 is a map illustrating the study area of interest which includes the surface water at up- and downstream sites of the Wonderfonteinspruit (WFS) and Mooi River (MR) confluence. This specific area supports a diverse range of anthropogenic activities impacting the water quality. The WFS flows from the Gauteng province, for 90 km, through one of the world’s richest gold mining fields (Coetzee et al. 2006) where it ultimately converges with the MR just after passing through the towns of Carletonville and Welverdiend. The MR flows in a north–south direction through, Klerkskraal-, Boskop- and Potchefstroom Dams (van der Walt et al. 2002). The MR is the major source of drinking water to Potchefstroom and a source of irrigation and livestock watering to farms surrounding the river. Before the confluence of the WFS with the MR, the MR is mainly impacted on by agricultural run-off, small informal settlements and limited mining activities (Coetzee et al. 2006). Six sites surrounding the MR and WFS confluence were selected. The site MR-BC 1 is least impacted by anthropogenic activities and served as reference site. Sites at Tlokwe municipality (now JB Marks Municipality) where the metal concentration relevant to the study were measured (Table 2) are also indicated in Fig. 1.

**Table 2:** Minimum co-selective concentrations (MCC; Seiler and Berendonk 2012) of relevant metals and the measured concentrations by the Tlokwe Municipality at sites in the Mooi River catchment on relevant dates. All values are in µg/L

	MCC	WFS		MR		GMB		BDI		BDC		PD	
Metals		March	July	March	July	March	July	March	July	March	July	March	July
Cu	0.03	28.0	1.0	18.0	2.0	8.0	32.0	5.0	31.0	3.0	27.0	3.0	12.0
Fe	NA	50.0	10.0	40.0	0.0	10.0	40.0	40.0	10.0	20.0	20.0	20.0	0.0
Pb	0.29	2.0	53.0	1.0	3.0	1.0	1.0	1.0	62.0	1.0	1.0	1.0	2.0
Zn	19.61	N/D	70.0	N/D	210.0	N/D	250.0	N/D	190.0	N/D	50.0	N/D	60.0

The March and July sampling dates of the Tlokwe dates were 10/03/ and 28/07/15 respectively. *WFS*, Wonderfonteinspruit; *MR*, Mooi River site; *GMB*, Gerhardminne Bron; *BDI*, Boskop Dam Inflow; *BDC*, Boskop Dam Canal; *PD*, Potchefstroom Dam; *N/A*, not applicable; *N/D*, none determined. *Cu*, copper; *Fe*, iron; *Pb*, lead; *Zn*, zinc

### Sample collection and physico-chemical analysis

Surface water samples were collected in sterile 1L glass containers according to the South African National Standard ISO 5667–6:2005 (SANS 2006). Sampling was conducted in March and July of 2015. March was a warm and wet period and July represented a cold and dry period, thus representing two seasons. Triplicate in situ measurements of the physical parameters temperature, pH and total dissolved solids (TDS) were measured with a Multi-Parameter Testr 35 Series (Eutech Instruments, Singapore) probe. Dissolved oxygen (DO) levels were measured using a Series 150 Multi-parameter (Orbeco Hellige, E-Chem instrument). Samples were placed on ice in sealed cooler boxes until further analysis (within 6 h). Duplicate measurements of the chemical parameters (phosphates; sulphates; nitrates; and chemical oxygen demand) were measured. Results were expressed as milligrams per litre (mg/L) according to appropriate methods and instructions set out by the manufacturer using a HACH DR 2800™ instrument and reagents (HACH, US).

### Enumeration of HPC bacteria resistant to specific antimicrobials

Serial dilutions of the water samples for each site were prepared and the total HPC bacteria were enumerated as described by Jordaan and Bezuidenhout (2016). R2A media supplemented with different antimicrobials were used to determine the presence of- and to enumerate antimicrobial resistant bacteria at each of the sampling sites. All stock solutions were prepared as described by Andrews (2001). Briefly, the stock solutions were sterilized through a 0.22 μm syringe filter into a sterile falcon tube and thereafter added to autoclaved and cooled (± 60 °C) R2A media. Aliquots (100 μL) of the dilution series were spread plated onto R2A-ampicillin plates (100 µg/mL) to screen for beta-lactam resistant HPC bacteria. The same process was repeated on R2A agar supplemented with the following metals (final concentration of 1 mM) individually: copper, iron, lead and zinc. The latter are heavy metals found in coal-fire gas minerals and are representative of heavy metals identified as by-products that are associated to South African mining activities (Ericson 1991; Pone et al. 2007). These were incubated according to the manufacturer’s instructions at room temperature for 5 days after which the CFU/mL were determined.

### Antibiotic susceptibility characterization of morphologically distinct isolates

Morphologically distinct isolates were selected from the antimicrobial-containing plates and sub-cultured on R2A supplemented with the same relevant antimicrobial from which the isolate originated. This was repeated at least 3 times. Gram-staining was performed according to Bergey (1994) on each isolate to determine the purity thereof.

A modified version of the disc diffusion method (Kirby et al. 2004) was used to determine the antibiotic susceptibility patterns. Briefly, overnight cultures were prepared in sterile Mueller Hinton Broth (Merck, Germany) and incubated at 26** °C**. One hundred microliter of each culture was spread aseptically onto Mueller Hinton Agar (Merck, Germany). Antibiotic discs for each of the antibiotics tested were placed aseptically onto spread plates and the plates were incubated at 26** °C** for 3–5 days. This was done in triplicate. Seven antibiotics from six antibiotic classes were included of which all were broad-spectrum antibiotics. The antibiotics included ampicillin (10 mg), amoxicillin (10 mg), tetracycline (30 mg), erythromycin (15 mg), streptomycin (15 mg), trimethoprim (5 mg) and chloramphenicol (30 mg). All the discs were from Mast Diagnostics (UK). After incubation, the inhibition zones were measured and recorded.

### Molecular identification of multi-resistant isolates

Morphologically distinct isolates from the total pool of multi-resistant isolates (resistant to three or more antibiotic classes) were selected for identification by 16S rRNA gene sequencing. DNA extraction was performed according Jordaan and Bezuidenhout (2016). Briefly, an overnight culture of each pure isolate was prepared in nutrient broth. Of this, 1.5 mL was centrifuged and the supernatant discarded. The pellet was re-suspended in 10 µL MiliQ water and microwaved at 1000 W for 2 min. Thereafter the mix was centrifuged at 13,400 rpms for 90 s and placed on ice. One microliter of the supernatant was used as DNA template in the PCR reaction mix. PCR amplification was performed as described by Jordaan and Bezuidenhout (2016). The successes of the PCRs were evaluated by electrophoresis on 1% agarose gels (w/v) (Jordaan and Bezuidenhout 2016). Amplified DNA fragments were sequenced by Inqaba Biotech (South Africa, Pretoria) and chromatograms of the sequences were visualized by Geospiza Finch TV (version 1.4) software. BLASTN searches (http;//www.ncbi.nlm.nih.gov/BLAST) were performed on all the amplified DNA sequences to identify the closest match with bacterial species in the GenBank database. All sequences were submitted to the Genbank database under accession numbers: MT993423-MT993457.

### Minimum inhibitory concentrations (MIC) of antimicrobials

A concentration gradient of antimicrobials supplemented to R2A media plates were prepared (as described previously) for each antimicrobial tested. Five concentrations (1.0 mM, 1.5 mM, 2.5 mM, 5.0 mM and 6.5 mM) were used for all the metal MIC assays. For the antibiotics (ampicillin, tetracycline, erythromycin and streptomycin) the following concentrations of each individual antibiotic was used: 50 mg/L, 100 mg/L, 130 mg/L, 150 mg/L, 180 mg/L and 200 mg/L. Pure colonies of morphologically distinct isolates were aseptically inoculated onto each concentration of the metal it was originally isolated from, and on the relevant antibiotics it had previously shown resistance to. Each isolate was also inoculated onto R2A without antimicrobial to serve as a non-selective control. Plates were incubated for 36–48 h at 26** °C**. After incubation-inoculated spots were evaluated for growth. The MIC was then assigned to be between the highest concentration on which growth was observed and the first concentration where there was no growth or potentially values higher than the maximum concentration.

### Statistical analysis

All averages were performed using Microsoft XL. The relationship of the physico-chemical and HPC results were analyzed by redundancy analysis performed by using CANOCO for Windows 4.0 (Ter Braak and Smilauer 1998) and visualized by a correlation bi-plot with a 5% significance level. Agglomerative hierarchical cluster analysis (AHC) was performed on multiple antibiotic resistance phenotype data using XLSTAT (v 2013.5.00). For the AHC analysis, each isolate was given a score of 1 if it was resistant (by disc diffusion method) or 0 if it was intermediate or susceptible to each antibiotic, individually, in order to detect the dissemination of antibiotic resistant phenotypes among sampling sites and selective media.

## Results

### Physico-chemical and antimicrobial-resistant HPC results

Averages of the physico-chemical quality, HPC counts and HPC-antimicrobial resistant surface water measurements are summarized in Table 1. Site MRBC 2 was dry during the July sampling period and therefore not included in the table. River temperatures recorded in March ranged between 20.5 and 27.8°C, whereas in July between 11.0 and 14.9 ℃. This is indicative of the sampling period. pH varied between 7.50 and 8.50 during both seasons. The total dissolved solids (TDS) consistently, with the exception of site MR-BC 1, exceeded the RWQO (370 mg/L; Table 1). Dissolved oxygen was generally lower in March (6.30 to 7.43 mg/L) compared to July (8.33 to 14.87 mg/L). Nitrate concentrations in the MR consistently exceeded the RWQO (0.30 mg/L) with some concentrations of up to 51.25 mg/L recorded at MR-BC 2. Phosphates were included for July and concentrations (0.34 to 7.24 mg/L) also exceeded the RWQO (0.40 mg/L) at all sites except MR-AC 1. The highest phosphate concentrations (3.40 to 7.43 mg/L) were recorded in the WFS sites. Sulphate levels were generally higher (109.5 to 150.00 mg/L) during the July period compared to March (1.50 to 90.5 mg/L), consistently exceeding the RWQO (75.0 mg/L) at both the WFS and the two MR-AC sites (> 85 mg/L).

Heterotrophic plate count (HPC) levels on un-supplemented R2A media ranged between 6.66 × 10^2^ and 2.75 × 10^6^ CFU/mL. The levels of these bacteria were one or more logs lower in July compared to March. Heterotrophic plate count bacteria were also found on all the antimicrobial (ampicillin, copper, iron, lead and zinc) containing R2A media. This was the case for most sites on both occasions. In March, MR-BC 2 had the highest number of resistant HPC bacteria to all the antimicrobials. Both WFS sites had higher levels of antimicrobial resistant bacteria compared to the MR sites. All sites, except MR-BC 1 in March, had ampicillin, iron and lead resistant bacteria on both occasions. Overall, the HPC bacteria were most susceptible to copper with levels ranging between 0.00 and 2.83 × 10^3^ on 1 mM Cu plates. No Cu-tolerant bacteria were detected at WFS 2 (July) and both MR sites after the confluence. The HPC were least susceptible to lead with levels ranging between 3.00 × 10^1^ and 2.80 × 10^5^ CFU/mL (Table 1).

**Fig. 2:**
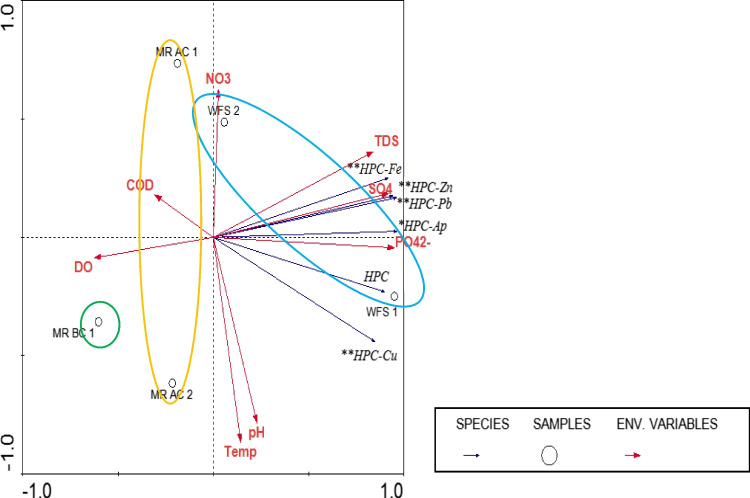
Redundancy analysis (RDA) correlation bi-plot indicating the statistical relationship between the average physico-chemical and HPC/antimicrobial resistant HPC results from five sites, surrounding the WFS and MR confluence of the overall averages of 2015. MR — Mooi River, WFS — Wonderfonteinspruit, BC — Before Confluence, AC — After Confluence, Temp — Temperature; TDS — total dissolved solids; DO — dissolved oxygen; COD — chemical oxygen demand; HPC — heterotrophic plate count, * HPC from antibiotic containing media. ** HPC from metal containing media.

Figure 2 displays a RDA bi-plot demonstrating the statistical relationship between average physico-chemical parameters and enumerated HPC bacteria, from 5 sites, for both occasions. The site MR BC 2 was eliminated from the analysis as the data were found to not be representative of only the water column but mixing of the sediment could have taken place. In the bi-plot, all the HPC counts from the antimicrobial containing plates had a close association to each other and also to SO_4_^2−^, TDS and PO_4_^2−^. However, the HPC counts on 1 Mm copper containing plates correlated weaker with the other HPC counts from antimicrobial containing media. DO and COD had a strong negative correlation with all of the HPC results and correlated best with the MR sites. The two WFS sites are situated on the right of the horizontal axis, closer to the HPC, SO_4_^2−^, TDS and PO_4_^2−^ grouping, whereas, the MR BC (control) site is situated far left, indicating opposite impacts. The two MR AC sites are situated between these opposite data points, indicating that they are influenced by both the MR BC and the WFS. The two WFS sites are located distant from one another, with WFS 1 presenting a strong positive correlation with the HPC, SO_4_^2−^, TDS and PO_4_^2−^ grouping and WFS 2 showing a strong positive correlation with NO_3_^−^_._ This suggests that a dilution effect of SO_4_^2−^, TDS and PO_4_^2−^ is present from the upstream to the downstream site. WFS 2 and MR AC 1 were closely grouped indicating that they have similar impacts. MR AC 2 and MR BC 1 grouped closer to one another suggesting that they are in a similar state; however, MR AC 2 is situated more to the right proposing that there is a greater presence of HPC, SO_4_^2−^, TDS and PO_4_^2−^ at this site compared to the control site.

### Percentage of isolates resistant to each antibiotic per site

**Fig. 3:**
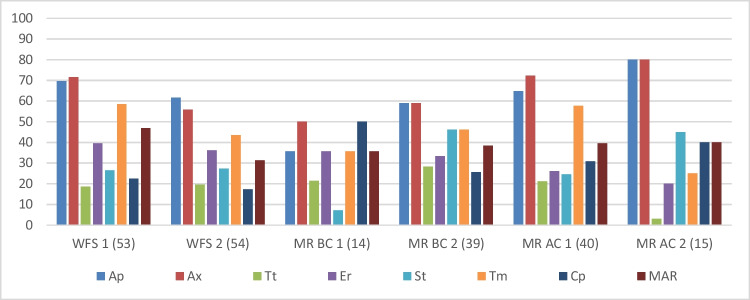
Bar chart of the percentage of total isolates, at each site, resistant to the individual antibiotics tested by the disc diffusion method**.** The numbers in brackets indicate the total amount of purified isolates that were tested against each antibiotic for the specific site. WFS — Wonderfonteinspruit, MR — Mooi River, BC — before confluence, AC — after confluence, Ap — ampicillin (10 µg), Ax — amoxicillin (10 µg), Tt — tetracycline (30 µg), Er — erythromycin (15 µg), St — streptomycin (20 µg), Tm — trimethoprim, Cp — chloramphenicol, MAR — multiple-antibiotic resistant

The bar chart in Fig. 3 illustrates the percentage of isolates, from the total number of isolates of both occasions that were resistant to the different antibiotics, per site. The number of isolates screened for antibiotic resistance per site, are indicated in the brackets next to the site name. A total of 215 isolates were screened for antibiotic resistance from both sampling occasions. Percentages for MR BC 1 and MR BC 2 sites were only representative of one sampling occasion at which isolates were detected. Results indicated the presence of resistance to all of the antibiotics screened for, at all of the sampling sites. More than 30% of the isolates at each site were multiple antibiotic resistant (MAR). The highest percentage of resistant bacteria were detected on the two beta-lactam antibiotics, followed by trimethoprim. Overall, the lowest percentage of isolates were tetracycline resistant.

### Agglomerative hierarchical cluster (AHC) analysis of multiple antibiotic resistant (MAR) phenotypes

**Fig. 4:**
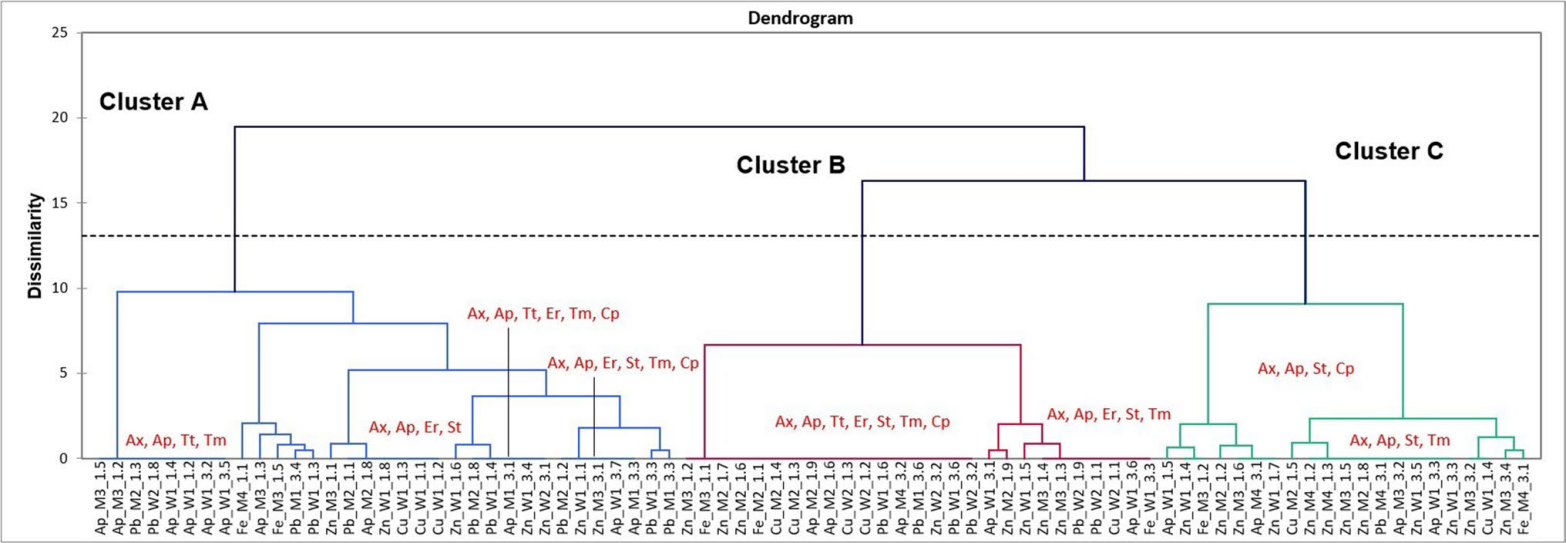
Dendrogram illustrating agglomerative hierarchical cluster analysis of multiple antibiotic resistant phenotypes of 81 isolates isolated from six sites surrounding the MR and WFS confluence, on media containing specific antimicrobials, on two sampling occasions in 2015**.** Ap — ampicillin (10 µg), Ax — amoxicillin (10 µg), Tt — tetracycline (30 µg), Er — erythromycin (15 µg), St — streptomycin (20 µg), Tm — trimethoprim, Cp — chloramphenicol, W1 — WFS 1, W2 — WFS 2, M1 — MR BC 1, M2 — MR BC 2, M3 — MR AC 1, M4 — MR AC 2

Figure 4 illustrates the similarity groupings of twenty seven MAR (> 3 classes of antibiotics) phenotypes (minor sub-clusters) from the 81 MAR isolates. Three branches are formed above the automatic truncation point calculated by the software, thus dividing the phenotypes into three clusters. These three clusters are further divided into sub clusters, which are divided into minor- sub clusters. The isolate label indicates the antimicrobial from which it was isolated, the site at which it was found, the sampling occasion and the sample number. A flat line on the dendogram indicates that more than one isolate had the specific phenotype. The eight most prevalent phenotypes are presented in Fig. 4. Eighteen isolates were resistant to all of the antibiotics screened for and evidently this was the most prevalent phenotype detected. Isolates exhibiting this phenotype were from all the antimicrobial containing media (ampicillin and the 4 metals) and all of the sampling sites. The 8 most prevalent phenotypes from both occasions in descending order, with regards to number of isolates, were as follows: Ap/Ax/Tt/Er/St/Tm/Cp > Ap/Ax/St/Tm > Ap/Ax/Tt/Tm > Ap/Ax/Er/St/Tm > Ap/Ax/Er/St > Ap/Ax/Tt/Er/Tm/Cp = Ap/Ax/Er/Tm/Cp > Ap/Ax/St/Cp. Each of the sub-clusters (Except the second sub-cluster in cluster A) represented at least one of the 8 most prevalent phenotypes, indicating that other minor-sub clusters found within the sub cluster had similar phenotypes to the prevalent phenotype found in that cluster.

### Resistance phenotypes and MICs of identified isolates

The identified isolates were positively identified as strains of the same species as their nearest similar sequence (16S rDNA) neighbour on the NCBI BLASTN database. Thirty-six multiple antibiotic resistant isolates were selected for identification based on morphology on the different media and from the different sites. They were identified to species (or at least genus) level belonging to 3 phyla (Proteobacteria, Bacteriodetes, Firmicutes; Table 3) of which most belonged to Proteobacteria. The majority of the identified Proteobacteria belonged to the Gammaproteobacteria class. Among these, there were 11 identified as species of the *Pseudomonas* genus and one isolate was a *Cellvibrio* sp. from the Pseudomonadaceae family. Ten isolates belonged to the genus *Acinetobacter* of the Moraxellaceae family. Two *Klebsiella pneumonia* isolates were identified as well as one *Serratia marcescens* from the Enterobacteriaceae family. Finally, one *Xantomonas* sp. and two *Stenotrophomonas maltophilia* isolates represented the Xantomonadaceae family. These species were from almost all the sampling sites (exception was MR-BC 1) and represented 11 of the multiple antimicrobial resistant phenotypes. Among the Alphaproteobacteria there were representatives of the *Rhizobium*, *Sphingobium*, *Sphingomonas* and *Agrobacterium* genera that were from the WFS 1, MR BC 2 and MR AC 1 sites. The Bacteriodetes phylum had representatives of the Flavobacteria and Sphingobacteria classes at the MR-AC 1 site. Five identified isolates from the two WFS and the two after confluence (MR) sites belonged to the Firmicutes phylum, with species belonging to the *Bacillus*, *Staphylococcus* and *Enterococcus* genera. Among the species that were originally isolated from metal containing media, MIC values ranged between 1.0 and > 6.5 mM and all of the isolates isolated from ampicillin had an MIC of over 200 mg/L for this antibiotic. A large proportion of isolates were resistant to the β-lactam antibiotics as well as tetracycline, erythromycin and trimethoprim. What is evident is that among these β-lactam resistant bacteria, whether they were originally isolated from ampicillin containing or metal containing media, they consistently had extremely high MICs to amoxicillin (> 100 mg/L). Extremely high MICs to erythromycin were observed among most of the *Gammaproteobacteria*. Moderate to high tetracycline MICs were also observed (Table 3).

**Table 3:** Antimicrobial resistance profiles of isolated and identified multiple antibiotic resistant bacteria

Identified species	Site of isolation	Antibiotic resistant phenotype	Isol. AM	MIC to Isol.Am*	MIC to different antibiotics (mg/L)			
					Ap	Tt	Er	St
***Proteobacteria***								
***Gammaproteobacteria***								
*Pseudomonas mosselii*	MR BC 2	Ax, Ap, Tt, Er, St, Tm, Cp	Ap	> 200	> 200	50–100	> 200	< 50
*Pseudomonas moraviensis*	WFS 2	Ax, Ap, Tt, Er, St, Tm, Cp	Pb	2.5–5.0	> 200	50–100	> 200	50–100
*Pseudomonas extremorientalis*	WFS 2	Ax, Ap, Tt, Er, Tm, Cp	Zn	5.0–6.5	> 200	50–100	> 200	-
*Pseudomonas gessardii*	WFS 1	Ax, Ap, Tt, Er, Tm, Cp	Zn	2.5–5.0	> 200	-	> 200	-
*Pseudomonas putida*	WFS 1	Ax, Ap, Tt, Er, Tm, Cp	Pb	2.5–5.0	> 200	50–100	> 200	-
*Pseudomonas putida*	MR BC 2	Ax, Ap, Tt, Tm	Pb	2.5–5.0	> 200	< 50	> 200	-
*Pseudomonas putida*	WFS 1	Ax, Ap, Er, Tm	Cu	1.5–2.5	> 200	-	50–100	-
*Pseudomonas rhodesiae*	WFS 1	Ax, Ap, Er, Tm	Cu	1.0–1.5	> 200	-	> 200	-
*Pseudomonas syringae*	MR BC 2	Ax, Ap, Er, Tm	Pb	2.5–5.0	> 200	-	> 200	-
*Pseudomonas composti*	MR AC 1	Ax, Ap, St, Tm	Pb	1.0–2.5	> 200	-	-	< 50
*Pseudomonas cichorii*	WFS 1	Ax, St, Tm	Cu	1.0–1.5	> 200	-	-	< 50
*Cellvibrio* sp.	WFS 1	Ax, Ap, Tt, Tm	Ap	> 200	> 200	< 50	-	-
*Acinetobacter beijerinckii*	MR BC 2	Ax, Ap, Tt, Er, St, Tm, Cp	Zn	5.0–6.5	> 200	130–150	> 200	50 -100
*Acinetobacter beijerinckii*	MR BC 2	Ax, Ap, Tt, Er, St, Tm, Cp	Cu	2.5–5.0	> 200	100–130	> 200	< 50
*Acinetobacter haemolyticus*	MR BC 2	Ax, Ap, Tt, Er, St, Tm, Cp	Cu	1.0–1.5	180–200	50–100	> 200	< 50
*Acinetobacter haemolyticus*	WFS 2	Ax, Ap, Tt, Er, St, Tm	Cu	1.0–1.5	> 200	50–100	> 200	< 50
*Acinetobacter haemolyticus*	WFS 1	Ax, Ap, Er, Tm	Cu	1.5–2.5	> 200	-	> 200	-
*Acinetobacter calcoaceticus*	MR BC 2	Ax, Ap, Tt, Er, Tm, Cp	Pb	5.0–6.5	100–130	< 50	< 50	< 50
*Acinetobacter* sp.	WFS 1	Ax, Ap, Tt, Er, St, Tm	Ap	> 200	> 200	50–100	> 200	> 200
*Acinetobacter* sp*.*	WFS 2	Ap, Tt, Er, St, Tm	Zn	2.5–5.0	100–130	50–100	> 200	< 50
*Acinetobacter* sp.	WFS 1	Ax, Ap, Tt, St, Tm	Zn	2.5–5.0	100–130	< 50	-	< 50
*Acinetobacter johnsonii*	WFS 1	Ax, Ap, Er, Tm	Ap	> 200	> 200	-	> 200	-
*Klebsiella pneumoniae*	WFS 1	Ax, Ap, Tt, Tm	Zn	1.5–2.5	130–150	> 200	50—100	-
*Klebsiella pneumoniae*	WFS 1	Tt, Er, Tm	Pb	2.5–5.0	-	50–100	< 50	-
*Serratia marcescens*	MR AC 1	Ax, Ap, Tt, Er, Tm	Zn	1.5–2.5	> 200	< 50	> 200	-
*Xantomonas* sp.	MR AC 1	Ax, Ap, Tt, Tm	Ap	> 200	> 200	< 50	-	-
*Stenotrophomonas maltophilia*	MR BC 2	Ax, Ap, Tt, Er, St, Tm, Cp	Ap	> 200	> 200	> 200	> 200	> 200
*Stenotrophomonas maltophilia*	WFS 2	Ax, Ap, Tt, Er, St, Tm	Zn	2.5–5.0	> 200	100–130	> 200	< 50
***Alphaproteobacteria***								
*Cand. Rhizobium massilae*	MR BC 2	Ax, Ap, Tt, Er, St, Tm, Cp	Zn	1.0–1.5	> 200	< 50	< 50	< 50
*Sphingobium yanoikuyae*	WFS 1	Ax, Ap, Er, St, Tm	Cu	1.5–2.5	> 200	-	< 50	< 50
*Rhizobium mongolense*	MR AC 1	Er, St, Tm	Pb	1.0–2.5	-	-	< 50	< 50
*Sphingomonas aerolata*	MR BC 2	Ap, St, Tm	Cu	2.5–5.0	> 200	-	-	< 50
*Agrobacterium vitis*	MR AC 1	Tt, Tm, Cp	Fe	1.5–2.5	-	< 50	-	-
***Bacteriodetes***								
***Flavobacteria***								
*Flavobacterium* sp.	MR AC 1	Ax, Ap, St, Tm	Ap	> 200	180–200	-	-	< 50
***Sphingobacteria***								
*Sphingobacterium* sp	MR AC 1	Ax, Ap, Tt, St, Cp	Fe	5.0–6.5	> 200	< 50	-	< 50
*Chitinophaga* sp.	MR AC 1	Ax, Ap, Tt, Er, St, Tm	Zn	2.5–5.0	> 200	< 50	> 200	50–100
***Firmicutes***								
***Bacilli***								
*Bacillus cereus*	MR BC 2	Ax, Ap, Tt, Er, St, Tm, Cp	Fe	1.5–2.5	> 200	> 200	> 200	> 200
*Bacillus thuringiensis*	MR BC 2	Ax, Ap, Er, Tm, Cp	Pb	5.0–6.5	> 200	-	< 50	-
*Staphylococcus* sp.	WFS 2	Ax, Ap, Tt, Er, St, Tm	Pb	2.5–5.0	> 200	100–130	> 200	50–100
*Enterococcus hirae*	MR AC 1	Ax, Ap, Tt, Er, St, Tm, Cp	Pb	2.5–5.0	> 200	50–100	> 200	50–100
*Enterococcus hirae*	WFS 1	Ax, Ap, Er, Tm, Cp	Zn	2.5–5.0	> 200	-	50–100	-

The site of isolation, the original supplemented media from which these were isolated as well as MIC values for the original antimicrobial substance as well as MIC values for 4 different antibiotics are also provided

* Unit for Ampicillin MIC is mg/L and for the metals mM, *MIC*, Minimum inhibitory concentration; *Isol.AM*, Isolation antimicrobial; *Cand*., Candidatus; *WFS*, Wonderfonteinspruit; *MR*, Mooi River; *BC*, Before confluence; *AC*, After confluence; *Ap*, Ampicillin; *Ax*, Amoxicillin; *Tt*, tetracycline; *Er*, Erythromycin; *St*, Streptomycin; *Tm*, Trimethoprim; *Cp*, Chloramphenicol

## Discussion

The aim of this study was to investigate antimicrobial resistance among heterotrophic plate count (HPC) isolates from surface water sites in the Mooi River and Wonderfonteinspruit system. There were two sites with no recorded gold mining impacts (MR-BC 1 and 2), two sites with direct gold mining impacts (WFS 1 and 2) and two sites in the Mooi River after the confluence of the two tributaries (MR-AC 1 and 2). Overall, HPC levels were lower during the colder months when the water temperature was cooler. This is similar to the findings of Ley et al. (2020). Additionally, studies by Villa et al. (2015), Valverde et al. (2015) noted that the growth of HPC bacteria is best observed at temperatures ranging between 20 and 30 °C. According to Jordaan and Bezuidenhout (2016), the decrease of HPC bacterial levels in colder temperatures may be attributed to temperature-induced stress that is responsible for the reduction of bacterial growth rates and survival of certain species. Furthermore, high temperature, NO_3_^−^, TDS, COD, and antimicrobial-resistant HPC levels were observed at site MR-BC 2 during the warm-wet season. On the day of sampling, cattle were grazing close to this site and the water level was low. It is possible that the cattle may have disturbed the sediment. The latter activity could have released nutrients and bacteria into the water column (Line et al. 1998). Additionally, several studies have indicated that decomposing organic matter can result in elevated water temperatures and COD levels (Awomeso et al. 2010; Li et al. 2020). Additionally, Sokolov et al. (2020) reported elevated COD levels in sites of high organic matter content. According to Garcia-Armisen et al. (2014); de Assis Costa et al. (2018) organic matter pollution can affect the self-purification process of a water body. Furthermore, studies by Aiken et al. (2011) illustrated that organic matter can contribute to the transport of pollutants such as heavy metals. Thus, the elevated temperature, COD, TDS, nitrate, and antimicrobial resistant HPCs could also be a result of organic matter pollution that may have occurred, at this site, prior to sampling. Also, the elevated nutrient and COD levels at site MR-BC 2, were not disseminated to downstream sites, this also strongly suggests that this was a site-specific occurrence and that the speculated disturbance or organic matter pollution did not have a downstream effect. Lastly, due to the drought experienced in this region, this site was dry during the July sampling run, and comparative data could not be made available. Furthermore, HPC on supplemented media were closely associated with each other and SO_4_^2−^, TDS and PO_4_^2−^. Patel and Parikh (2013) note that increased TDS levels propagate potentially pathogenic bacterial levels thus decreasing the quality of the water. Also, the elevated TDS and SO_4_^2−^ levels at the MR sites after the confluence when compared to the sites before the confluence suggested that the mining pollution of the WFS has a definite influence on the water quality of the receiving downstream MR sites.

The results of the current study demonstrated that antimicrobial-resistant bacteria were present at all the sites of interest. Additionally, ampicillin-resistant bacteria were abundant at all sites and ranged from 10^1^ to 10^3^ cfu/mL during both sampling periods. Similar results were observed by Henriques et al. (2016). These authors investigated the co-selection of metal and antibiotic resistance in the epiphytic bacterial community in contaminated salt marches. They found that of all the antibiotics they tested on metal-resistant bacteria, from contaminated sites, the occurrence of ampicillin and amoxicillin resistance was most abundant. The review by Squandrone (2020) supports the latter as it depicted numerous studies that reported co-resistance between metals and the beta-lactam group in aquatic studies. However, evidence (Fig. 2) is provided that a large percentage of the ampicillin and metal-tolerant HPC bacteria were also resistant to other antibiotic classes suggesting the co-occurrence of metal and antibiotic resistance. The anthropogenic impacted sites (WFS, MR sites BC 2 to AC1 and 2) depicted high antimicrobial-resistant bacteria loads compared to the minimal impacted site (MR-BC 1). This trend was also observed by various other studies investigating different metal-polluted aquatic environments (Naik et al. 2013; Chudobova et al. 2014; Pal et al. 2015; Wales and Davies 2015; Henriques et al. 2016; Truong et al. 2021). These studies all concluded that environmental antimicrobial pollution (i) selects for resistance to specific antimicrobials and (ii) it may co-select for multiple resistance to a range of antibiotics. Additionally, the most prevalent resistance phenotypes (included all antibiotic classes: Ap/Ax/Tt/Er/St/Tm/Cp) were found more abundantly in isolates isolated from media that were supplemented with metals (Table 3) compared to the ampicillin media isolates. This indicates that the metal pollution in the area of the current study is not only co-selecting for one or two antibiotics but an entire range of antibiotic classes and therefore present health risks (Chudobova et al. 2014). The findings of this study support the suggestions that heavy metals, due to their toxic effects, have the ability to alter both microbial community structures and microbial activities (Giller et al. 2009; Shuaib et al. 2021). Squandrone (2020) explains that these metal antibiotic co-selections are possibly catalysed by Intergrons which acquire and exchange gene cassettes as well as class Intergrons that have readily been identified in contaminated environments. More so, the results of Zhang et al. (2020) demonstrate that bacterial communities react to the type of metal shock they are exposed to. Additionally, the resistance of the latter bacteria to antibiotics becomes enhanced, consequently activating various classes of ARGs, intergrons, and transposons. Thus, modifying the antibiotic resistome. This mechanism provides bacteria a selective advantage when exposed to unfavourable conditions (Seiler and Berendonk 2012).

In the current study, resistance was assigned conservatively. Almost all the metal resistant isolates were also tolerant to one or more antibiotic class, indicating that there is a co-occurrence of resistance to metal and antibiotic resistance. Similar findings have been reported in other studies (Chen et al. 2015; Tsvetanova et al. 2022). Also, recent studies have provided evidence that a large percentage of the ampicillin and metal tolerant HPC bacteria are also resistant to other antibiotic classes (Çiftçi Türetken et al. 2019; Squandrone 2020). Resistance to the two β-lactam antibiotics (Ampicillin/Amoxicillin) was found most frequently among the isolates. The abundance of β-lactam resistance determinants present in the natural environment and its relationship to metal resistance has also been demonstrated in a number of previous studies (Henriques et al. 2016; Luczkiewicz et al. 2015; Chudobova et al. 2014; Allen et al. 1977). In the current study, tetracycline resistance was less common since only a limited number of isolates were resistant to this antibiotic. This contrasts with results reported in other studies where tetracycline resistance was ubiquitous among the metal tolerant bacteria (Chen et al. 2015; Okugbe et al. 2019). This suggests that the co-selection of metal and antibiotic resistance is complex and different factors influence the phenomenon. Furthermore, more than a third of the antimicrobial tolerant isolates from all of the sites, displayed MAR. This indicates that pollution around the current study is not only co-selecting for one or two antibiotics, but an entire range of antibiotic classes and therefore, present health risks especially for the immunocompromised (Chudobova et al. 2014; Mulamattathil et al. 2014; Baker-Austin et al. 2006). Similar results were found in a previous study (Chudobova et al. 2014).

The most prevalent resistance phenotype (Ax, Ap, Tt, Er, St, Tm, Cp) was found more abundantly among isolates from media that were supplemented with metals compared to the isolates enumerated from ampicillin containing media. Furthermore, the concentrations of Cu, Pb and Zn measured in the surrounding area of the current study exceeded the MCC levels. This supports the assumption that metal pollution is a potential driver of the co-occurrence of metal and antibiotic resistance in the current study. Copper is commonly used as a growth promoter and therapeutic agent for livestock (Wales and Davies 2015; Sharif et al. 2021). The high abundance of Cu tolerant HPC bacteria at these sites could thus potentially be ascribed to livestock farming in the vicinity of the sampling points (van der Walt et al. 2002) and that the copper pollution at the WFS sites could be due to both mining impacts and agricultural impacts. For the MR-BC 2 site, pollution was most probably due to only agricultural impacts and especially animal manure that have settled in the sediment and then re-suspended into the water column by cattle (Tuckfield and McArthur 2008). Studies have reported the presence of Cu as a feed additive in farming (Cannatelli et al. 2014; Yazdankhah et al. 2014). Thus, the sediment may carry more antibiotic-resistant bacteria than the water column. Furthermore, the largest loads of potentially metal-tolerant HPC bacteria were enumerated on lead (Pb) containing media. This high tolerance to Pb might be attributed to the low solubility of the metal and ultimately low bioavailability (Nies 1999). However, Pb resistance should not be ignored as a possible driver for antibiotic resistance in the current study as the concentration for Pb exceeded the MCC values set out by Seiler and Berendonk (2012). Many P-type ATPase efflux systems have been associated with plasmid encoded Pb resistance (Nies 1999; Naik et al. 2013). Drudge et al. (2012) found a Pb resistance gene cluster alongside genes encoding multiple antibiotic resistance on transferable plasmids in floc bacteria influenced by many trace elements. The authors (Drudge et al. 2012) explained that the presence of trace elements activates the SOS response in bacteria. This could stimulate the exchange of gene cassettes. Thus, plasmids, transposons and integrons could be commonly disseminated in the bacterial community during stress conditions leading to rapid spread of multiple resistance determinants. Zinc (Zn) tolerant bacteria were enumerated from all sites except MR-BC 1 and MR-AC 2 (July). This metal is common in sulphate ores and elevated concentrations measured may be due to release by mining activities (Spitz and Trudinger 2019). Animal manure may also contain high concentrations of heavy metals such as Zn and therefore they may accumulate in the sediment over time (Zhang et al. 2012; Xue et al. 2021; Nakagawa et al. 2022). Furthermore, Zn has a high affinity for organic matter in sediment (Seiler and Berendonk 2012). Our results showed very high levels of Zn tolerant HPC bacteria at MR-BC 2 (4.15 × 10^4^ CFU/mL). At the other sites, the levels were 2 to 3 log lower. This is the same site that was potentially impacted by cattle disturbing it just prior to sampling. Interestingly, Cu and Zn tolerant HPC bacteria were detected at WFS 1, 2 and MR-BC2. According to Glibota et al. (2019), environmental studies have illustrated a related correlation between these two metals and the presence of metal-tolerant and antibiotic-resistant microbial populations. This combined tolerance has previously been reported and is ascribed to genetic elements harbouring both metal and antibiotic resistance genes (Máthé et al. 2012; Gullberg et al. 2014; Poole 2017).

The presence and resistance patterns of these species isolated and characterized could thus have health implication as the Mooi River is used for recreation as well as agricultural purposes. Treatment of infection caused by such pathogenic/opportunistic pathogens in consumers in general and especially the immunocompromised will be challenging (Jordaan and Bezuidenhout 2016).

## Conclusions

The current study is contributing to understanding the potential compounded impacts of metals and antibiotics from mining, agriculture and urbanization on heterotrophic bacteria in the aquatic environment in gold mining impacted aquatic systems of the Gauteng and North West Provinces of South Africa. The results demonstrated that gold mining activities in the WFS have a detrimental impact on the downstream physico-chemical and bacteriological quality of water to the rural communities around the area of interest.

This is a very complex phenomenon and an in-depth molecular study on the specific determinants present in the bacteria will be critical for a better understanding of the mechanisms driving the potential co-selection processes. Furthermore, metagenomics and meta-transcriptomics should be incorporated to provide better insights in genetic determinants present and their expression in the entire bacterial population of the study area. The study did, however, demonstrate that different anthropogenic activities surrounding the system are degrading the quality of the water served to the downstream town of Potchefstroom and that there is a co-occurrence of metal and antibiotic resistant bacteria present in the water. Furthermore, the current situation raises concerns about human and animal health.
